# Differences in the transcriptome signatures of two genetically related *Entamoeba histolytica *cell lines derived from the same isolate with different pathogenic properties

**DOI:** 10.1186/1471-2164-11-63

**Published:** 2010-01-26

**Authors:** Laura Biller, Paul H Davis, Manuela Tillack, Jenny Matthiesen, Hannelore Lotter, Samuel L Stanley, Egbert Tannich, Iris Bruchhaus

**Affiliations:** 1 Bernhard Nocht Institute for Tropical Medicine, Bernhard-Nocht-Str. 74, 20359 Hamburg, Germany; 2 Department of Biology, University of Pennsylvania, Philadelphia, Pennsylvania, USA; 3 Office of the President, Stony Brook University, Stony Brook, New York, USA

## Abstract

**Background:**

The availability of two genetically very similar cell lines (A and B) derived from the laboratory isolate *Entamoeba histolytica *HM-1:IMSS, which differ in their virulence properties, provides a powerful tool for identifying pathogenicity factors of the causative agent of human amoebiasis. Cell line A is incapable inducing liver abscesses in gerbils, whereas interaction with cell line B leads to considerable abscess formation. Phenotypic characterization of both cell lines revealed that trophozoites from the pathogenic cell line B have a larger cell size, an increased growth rate *in vitro*, an increased cysteine peptidase activity and higher resistance to nitric oxide stress. To find proteins that may serve as virulence factors, the proteomes of both cell lines were previously studied, resulting in the identification of a limited number of differentially synthesized proteins. This study aims to identify additional genes, serving as virulence factors, or virulence markers.

**Results:**

To obtain a comprehensive picture of the differences between the cell lines, we compared their transcriptomes using an oligonucleotide-based microarray and confirmed findings with quantitative real-time PCR. Out of 6242 genes represented on the array, 87 are differentially transcribed (≥two-fold) in the two cell lines. Approximately 50% code for hypothetical proteins. Interestingly, only 19 genes show a five-fold or higher differential expression. These include three *rab7 GTPases*, which were found with a higher abundance in the non-pathogenic cell line A. The *aig1-like GTPases*are of special interest because the majority of them show higher levels of transcription in the pathogenic cell line B. Only two molecules were found to be differentially expressed between the two cell lines in both this study and our previous proteomic approach.

**Conclusions:**

In this study we have identified a defined set of genes that are differentially transcribed between the non-pathogenic cell line A and the pathogenic cell line B of *E. histolytica*. The identification of transcription profiles unique for amoebic cell lines with pathogenic phenotypes may help to elucidate the transcriptional framework of *E. histolytica *pathogenicity and serve as a basis for identifying transcriptional markers and virulence factors.

## Background

The human protozoan parasite *E. histolytica *resides in the large bowel and can persist there for months or even years, causing asymptomatic luminal gut infection. Occasionally, *E. histolytica *trophozoites penetrate the intestinal mucosa, causing amoebic colitis and spread via portal circulation to other organs, most commonly to the liver, where they induce abscess formation.

Currently, the factors determining the clinical outcome of *E. histolytica *infection are unknown, although a number of different hypotheses have been made. Host or parasite genetic factors could play a role, but so could the nature of the immune response, as well as concomitant infections or even diet.

The mechanisms and processes that allow Entamoeba to penetrate the tissue of its host and induce colitis and liver abscesses are not completely understood. Several studies have dealt with the identification of pathogenicity factors of *E. histolytica*. In particular, the galactose/N-acetyl D-galactosamine-inhibitable (Gal/GalNAc) lectins, the cysteine peptidases and amoebapores have been related to pathogenicity (for review [1]). However, these molecules cannot exclusively be responsible for amoebic virulence, because they are found in pathogenic as well as in non-pathogenic *E. histolytica *isolates. Beside the characterization of individual molecules, different approaches comparing the transcriptomes or proteomes of virulent and non-virulent isolates have been performed. In nearly all of these studies, a comparison was made between the pathogenic *E. histolytica *isolate HM-1:IMSS and the non-pathogenic isolate Rahman [2-5]. HM-1:IMSS was isolated in 1967 from a patient with amoebic dysentery, whereas Rahman was originally isolated in England 1973, from an asymptomatic individual. In contrast to HM-1:IMSS, Rahman does not form liver abscesses in animal models and has a defect in causing amoebic colitis in human colonic xenografts. In addition, a reduced cytotoxicity *in vitro *for epithelial cellsand a defect in erythrophagocytosis was observed [6,7]. However, due to the high genetic variability of *E. histolytica *isolates, differences between pathogenic and non-pathogenic isolates might simply reflect inter-isolate variation rather than specific differences linked to virulence.

To circumvent the problem of substantial genetic inter-isolate variation, we have recently analyzed two cell lines derived from the *E. histolytica *isolate HM-1:IMSS, called cell line A and cell line B. Genetically, both share identity in the highly polymorphic tandem repeat DNA sequences tested, but cell line B consistently produces large liver abscesses in a gerbil model of disease, whereas cell line A does not [8].

Phenotypic analysis of both cell lines revealed in cell line A an increased hemolytic activity, a lower growth rate, smaller cell size, a reduced cysteine peptidase activity and a lower resistance to nitric oxide stress. Proteomic comparison by two-dimensional difference gel electrophoresis (2D-DIGE) followed by mass spectrometry, identified a total of 21 proteins with higher abundance in cell line A and ten proteins with higher abundance in cell line B. Notably, in the case of only two differentially regulated proteins, namely a Fe-hydrogenase 2 and a C2 domain protein, was a differential expression also found on the level of transcription [8].

Here we compare the transcriptomes of cell line A and cell line B to get a more complete picture of the biomolecular differences between these cell lines. For this we used a custom 70-mer oligonucleotide-based microarray, previously applied to compare the transcriptomes of Rahman and HM-1:IMSS [3].

Our data indicate that there are significant and reproducible transcriptional differences between these cell lines. Most differentially expressed genes belong to the family of small GTPases. These are of special interest as putative *E. histolytica *pathogenicity factors, because most members of the family coding for the AIG1-GTPases are upregulated in the pathogenic cell line B. On the other hand, some Rab GTPases were found in higher levels in the non-pathogenic cell line A. The determination of the specific expression profiles of the non-pathogenic cell line A and the pathogenic cell line B may help provide new insights into the mechanisms that have enabled *E. histolytica *to become a pathogen.

## Results

The aim of this work is to identify potential pathogenicity factors, by comparing the transcriptional profiles of two genetically related *Entamoeba histolytica *HM-1:IMSS cell lines with different virulence phenotypes.

Both cell lines are derived from the *E. histolytica *isolate HM-1:IMSS as both were originally obtained from the American Type Culture Collection (ATCC) under the catalogue number 30459. To examine whether the cells from the two cell lines are genetically related and indeed derived from isolate HM-1:IMSS, tRNA-linked short tandem repeat (STR) sequences from six different loci were analyzed [8].

When these cells are used for infection experiments to induce amoebic liver abscesses in gerbils, cell line A produces very small lesions of pin-head size only, whereas cell line B induces significant abscesses affecting up to 30% of the liver [8]. The pathogenic phenotype of cell line B appears to be a constant property as it has been maintained without any animal passage for at least five years. The stability of the phenotype was tested routinely every six month.

### Comparison of the transcriptomes of *E. histolytica *cell line A and cell line B

To identify differences between cell lines A and B on a molecular level, we compared their transcriptomes. For this study, a custom-made 70-mer oligonucleotide microarray was used containing 6242 unique genes found in the *E. histolytica *genome dataset of February 2004. The microarray data are deposited in the public database ArrayExpress http://www.ebi.ac.uk/arrayexpress under the accession number E-MEXP-2504.

Using this array and analyzing two biological replicates, 87 gene transcripts were detected that show a two-fold or greater difference in expression between cell line A and cell line B. Out of these, 47 genes were significantly upregulated in the non-pathogenic cell line A and 40 genes were transcribed at significantly higher levels in the pathogenic cell line B (additional file 1).

We used quantitative real-time PCR to confirm the differential transcription of 27 selected genes that showed at least a three-fold higher level of transcription in one or other cell line. For all 27 analyzed genes, the real-time PCR results matched the microarray data (additional file 1).

Of the 87 differentially transcribed genes, 39 could be classified by putative biological function, namely stress response, trafficking/targeting, transporter, signaling, kinases, RNA/DNA metabolism, cell cycle, cell metabolism, peptidases, lectins, and AIG1 family proteins. The remaining 48 genes were categorized as genes coding for proteins of unknown function in *E. histolytica *(additional file 1).

Altogether, 17 of the deduced proteins of the 87 identified differentially transcribed genes contain one to eleven transmembrane domains. Interestingly, of these, five have no predicted signal peptide or signal anchor sequence, including two of the four AIG1 transcripts identified (additional file 1).

The majority of the differentially transcribed genes show only a two- to four-fold difference in expression. These include 39 genes for cell line A and 29 genes for cell line B. Only a limited number of genes (19 in total) show a five-fold or higher differential expression between the cell lines, as determined by microarray analyses and/or real time-PCR. Due to their highly significant differential expression (1000-fold in some cases), and comparative observation of nearly all other transcripts in the parasite, it is likely that these identified molecules are involved in the large difference in virulence observed between cell lines A and B. Three of the highly differentially expressed genes are *rab7 gtpases*, which are transcribed in higher levels in cell line A. This was confirmed using quantitative real-time PCR. Here, the differential transcription was between 50- and 740-fold. In addition, two genes coding for C2 domain-containing proteins were also expressed in higher levels in cell line A. Like the Rab proteins, molecules containing a C2 domain may be involved in regulating membrane traffic pathways. Quantitative real-time PCR indicates that together with one hypothetical protein [GenBank:XM_649962], a C2 domain-containing protein shows the highest differential transcription (approximately 1000-fold) of all analyzed genes (Table 1, additional file 1).

**Table 1:** List of genes differentially transcribed (≥ 5-fold) in *E. histolytica *HM-1:IMSS cell lines A and B identified by microarray analyses and real-time PCR

Gene name	Accession-No. Gene	Microarry results (x-fold)		Real time-PCR results (x-fold)	
		**Cell line A**	**Cell line B**	**Cell line A**	**Cell line B**

EhRab7E protein	XM_646110	62.00		743.45	

Hypothetical protein	XM_648456	52.11		866.52	

EhRab7D protein	XM_646823	25.37		125.00	

EhMP8-2 (gp63)	XM_647540	21.44		70.58	

C2 domain containing protein	XM_650207	16.65		1000.00	

EhRab7G protein	XM_651385	14.50		50.00	

C2 domain containing protein	XM_650951	6.62		33.35	

Hypothetical protein	XM_644469	3.52		5.71	

Hypothetical protein	XM_643681	3.46		7.53	

AIG1 family protein	XM_648725		14.29		100.00

Hypothetical protein	XM_645291		14.28		50.00

AIG1 family protein	XM_645223*		12.50		4.76

Hypothetical protein (AIG)	XM_648115*		12.50		100.00

Hypothetical protein	XM_645139		9.09		5.00

Hypothetical protein	XM_647137		8.33		7.14

Serine-threonine-isoleucine rich protein	XM_648869		6.25		3.85

Hypothetical protein	XM_649962		5.26		980.00

Hypothetical protein	XM_646695		5.3		4.5

AIG1 family protein	XM_643009*		4.34		100.00

(Fe-hydrogenase 2	XM_647747		2.44		3.22)^#^

* Removed from NCBI

^# ^Identified as differential in proteomic and transcriptomic approach

Furthermore, transcript levels for a cell surface protease gp63 (*ehmp8-2*) [GenBank:XM_647540] are also found in much higher levels in cell line A compared to cell line B (Table 1). Within the *E. histolytica *genome, two *gp63 *genes are present, which are termed *ehmp8-1 *[GenBank: XM_650302] and *ehmp8-2*. These molecules show 34% identity to each other and contain both a signal peptide and a transmembrane domain. In contrast to *ehmp8-2*, the expression of *ehmp-8-1 *is similar in both isolates as indicated by microarray analyses and real-time PCR (data not shown).

Additionally, three transcripts representing hypothetical proteins were found in higher abundances in cell line A. One shows a 50-fold higher expression level in cell line A in comparison to cell line B. This gene encodes for a protein of 105 amino acids, and 80% of the protein consists of the amino acids Pro, Gly, Met, Tyr and Ala. The stretch Gyl-Ala-Tyr-Pro-Pro-Met is present four times within the sequence and homology searches indicates an approximate 50% identity to the N-terminal region of annexins.

In the pathogenic cell line B, six genes coding for hypothetical proteins could be identified that show a 5-fold or higher expression level in comparison to cell line A. For two of the respective proteins, a signal anchor as well as a transmembrane domain could be predicted (Table 1, additional file 1).

### Identification of the *E. histolytica *AIG1 protein family

**Figure 1:**
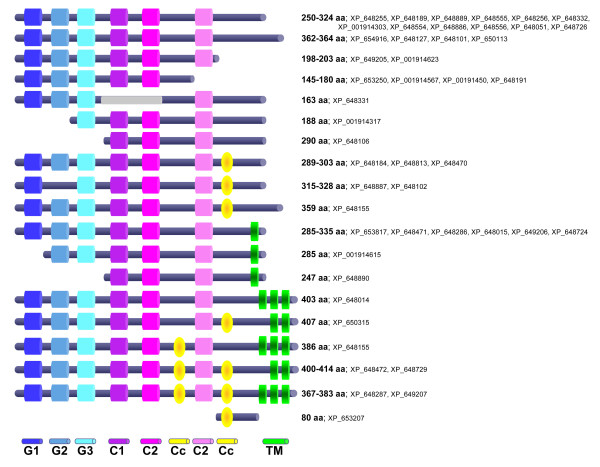
**Schematic description of the putative AIG-Proteins of *E. histolytica***. GTP-binding motifs: G1 = GxxxxGKS, G2 = SET; G3 = VIDTPGL; Conserved domains: C1 = GL/I/VQG/AIV/II/LV/TL/MN, C2 = HVCIVWTKC, C3 = RSExEIERLI; TM: predicted transmembrane domain; Cc: Predicted coiled-coil motif.

The oligonucleotide array that was used in this study covers about 75% of the annotated amoebic genes. It includes oligonucleotides for four putative *aig1-GTPase *genes, all expressed in higher levels in the pathogenic cell line B (Table 1, additional file 1). Basic Local Alignment Search Tool (BLAST) analyses indicated that the *E. histolytica aig1 *gene family consists of 47 members. The composed list of *aig1*genes includes 17 members where the RefSeq record was removed from NCBI as a result of standard genome annotation processes (Table 2). Nevertheless, some of the removed genes have been cloned in our laboratory [GenBank: XM_649824, XM_643009, XM_645223, XM_648115] and the respective sequences were also found in whole genome shotgun reads (*Entamoeba histolytica *HM-1:IMSS, taxid:294381). The *E. histolytica*AIG1 family members show structural similarity to the GTPases of immunity-associated protein (GIMAPS)/immune-associated nucleotide-binding protein (IAN) family of AIG1-like GTPases, which are conserved between vertebrates and angiosperm plants [9]. The members of this family comprise 30-80 kDa proteins, characterized by an AIG1 domain (a GTP-binding motif) and coiled-coil motifs. The GTP-binding motif is composed of the G1 to G5 sequences and two conserved motifs (CB, consensus box and IAN motif) [9]. The *E. histolytica *AIG1 family members have a calculated molecular mass between 20 and 45 kDa. Most of the amoebic AIG1 molecules have the first three of the five GTP-binding sites. The CB motif and the IAN motif are not present within the amoebic proteins; instead they contain three specific domains, which are conserved throughout the amoebic protein family. As described for the GIMAP molecules, some amoebic AIG1 proteins have one or two putative coiled-coil domains. In addition, there is a subgroup that contains one to three C-terminal transmembrane domains. Some of the GIMAP members also contain hydrophobic regions at the C-terminus, which are thought to be involved in membrane anchoring [9] (Table 2; Figure 1).

**Table 2:** List of AIG1-like family members

	GenBank Accession no. Gene	GenBank Accession no. Protein	Size aa	G1	G2	G3	C1	C2	C3	Cc** (position aa)	TM*** (position aa)	Real-time PCR data (x-fold)	
												**Cell line A**	**Cell line B**

**A > B**													

1*	XM_643035	XP_648127	364	+	+	+	+	+	+			12.5	

**A < B**													

2	XM_648725	XP_653817	335	+	+	+	+	+	+		307 - 324		100.00

3*	XM_643009	XP_648101	364	+	+	+	+	+	+				100.00

4*	XM_648115	XP_653207	80	-	-	-	-	-	-	Th**** 50%: 14 - 24			100.00

5*	XM_643380	XP_648472	400	+	+	+	+	+	+	Th 50%: 150 - 175 262 - 265	327 - 350 372 - 393		20.00

6*	XM_643637	XP_648729	414	+	+	+	+	+	+	Th 50%: 152 - 174 203 - 265	334 - 360 386 - 406		20.00

7	XM_643379	XP_648471	308	+	+	+	+	+	+		284 - 305		16.67

8	XM_644114	XP_649206	309	+	+	+	+	+	+		285 - 306		12.50

9	XM_642923	XP_648015	306	+	+	+	+	+	+		283 - 305		11.11

10*	XM_645021	XP_650113	364	+	+	+	+	+	+				11.11

11	XM_643798	XP_648890	247	-	-	-	+	+	+		211 - 232		10.00

12*	XM_649824	XP_654916	362	+	+	+	+	+	+				10.00

13*	XM_648140	XP_653232	359	+	+	+	+	+	+	Th 90%: 221 - 235			5.56

14*	XM_645223	XP_650315	407	+	+	+	+	+	+	Th 90%: 302 - 313	324 - 345 351 - 369		4.76

15	XM_643164	XP_648256	304	+	+	+	+	+	+				3.57

16*	XM_643099	XP_648191	180	+	+	+	+	+	-				2.70

17	XM_643163	XP_648255	324	+	+	+	+	+	+				2.94

18	XM_643194	XP_648286	314	+	+	+	+	+	+		290 - 311		2.63

19	XM_643240	XP_648332	294	+	+	+	+	+	+				2.63

**A = B**													

20*	XM_643464	XP_648556	304	+	+	+	+	+	+			1.25	

21*	XM_643462	XP_648554	289	+	+	+	+	+	+			1.22	

22*	XM_642922	XP_648014	403	+	+	+	+	+	+		288 - 309 315 - 341 352 - 374	1.12	

23	XM_643102 XM_001914071	XP_648194 XP_001914106	303	+	+	+	+	+	+	Th 90%: 227 - 251		1.06	

24*	XM_642959	XP_648051	291	+	+	+	+	+	+			1.04	

25	XM_643063	XP_648155	386	+	+	+	+	+	+	Th 90%: 148 - 167	286 - 313 316 - 341 348 - 372		1.92

26	XM_643097	XP_648189	298	+	+	+	+	+	+				1.89

27	XM_644113	XP_649205	198	+	-	+	+	+	+				1.79

28	XM_644115	XP_649207	383	+	+	+	+	+	+	Th 90%: 194 - 208 211 - 217	287 - 308 310 - 335 341 - 369		1.67

29	XM_643463	XP_648555	319	+	+	+	+	+	+				1.64

30*	XM_643100	XP_648192	328	+	-	+	+	+	+	Th 50%: 149 - 166			1.61

31	XM_643195	XP_648287	367	+	+	+	+	+	+	Th 90%: 150 - 169 196 - 233	286 - 313 315 - 336 341 - 365		1.54

32	XM_643795	XP_648887	315	+	-	+	+	+	+	Th 90%: 153 - 166			1.30

33	XM_643721	XP_648813	291	+	+	+	+	+	+	Th 50%: 195 - 206			1.27

34	XM_648158	XP_653250	178	+	+	+	+	+	-				1.01

**n.d.**													

35	XM_643797	XP_648889	287	+	+	+	+	+	+				

36	XM_643634	XP_648726	290	+	+	+	+	+	+				

37	XM_001914268	XP_001914303	298	+	+	+	+	+	+				

38	XM_643632	XP_648724	308	+	+	+	+	+	+		286 - 305		

39	XM_001914588	XP_001914623	203	+	+	+	+	+	+				

40	XM_001914580	XP_001914615	285	-	+	+	+	+	+		260 - 282		

41	XM_001914532	XP_001914567	145	+	+	+	+	+	-				

42	XM_643239	XP_648331	163	+	+	+	-	-	+				

43	XM_001914473	XP_001914508	179	+	+	+	+	+	-				

44	XM_001914282	XP_001914317	188	-	-	+	+	+	+				

45*	XM_643794	XP_648886	250	+	+	+	+	+	+				

46*	XM_643014	XP_648106	290	-	-	-	+	+	+				

47*	XM_643378	XP_648470	289	+	+	+	+	+	+	Th 50%: 212 - 241			

GTP-binding motifs: G1 = GxxxxGKS; G2 = SE**T**; G3 = VIDTPGL; G4 and G5 = not present; Conserved domains: C1 = GL/I/VQG/AIV/II/LV/TL/MN; C2 = HVCIVWTKC; C3 = RSExEIERLI; n.d.: not determined; *Removed from NCBI; **Prediction of coiled-coil domains using Marcoil1.0 http://www.isrec.isb-sib.ch/webmarcoil/webmarcoilC1.html[28]

***Prediction of transmembrane domains (TM) using TMHMM Server http://www.cbs.dtu.dk/services/TMHMM

****Treshold (Th)

### Transcription profiles of aig1 genes in cell line A and cell line B

Using quantitative real-time PCR, the transcription profiles of 34 *aig1 *genes were analyzed. Interestingly, 18 of these analyzed genes are transcribed at higher levels in the pathogenic cell line B, whereas only one gene was more highly transcribed in the non-pathogenic cell line A. For three *aig1 *genes, the transcription level was at least 100-fold higher, and for eight genes more than 10-fold higher in cell line B compared to cell line A. The transcription level of the remaining 15 genes was similar in the two cell lines (Table 2).

## Discussion

In order to identify transcripts that are involved in or even responsible for pathogenicity, we compared two highly related *E. histolytica *isolates that differ substantially in their pathogenicity. The *E. histolytica *HM-1:IMSS cell line B is highly pathogenic and produces large liver abscesses in an animal model, whereas HM-1:IMSS cell line A appears to have lost its ability to induce abscess formation [8].

The microarray applied in this study had already been used to identify the differences between the non-pathogenic isolate Rahman and the pathogenic isolate HM-1:IMSS [3]. Using this array, which covers 75% of *E. histolytica *genes, 1.4% of the analyzed genes showed a two-fold or greater difference in expression between cell line A and cell line B. Only 0.3% of the genes showed a five-fold and higher differential transcription.

Astonishingly, there is only a small overlap between our transcriptomic and proteomic studies that compare the two cell lines. Only two genes were fond to be differentially expressed both on a transcript and protein level; Fe-hydrogenase 2 [GenBank:XM_647747] at higher abundance in cell line B and one C2 domain protein [GenBank:XM_650207] at a higher abundance in cell line A [8]. A similar phenomenon was also observed when comparing the transcriptomes and proteomes of Rahman and HM-1:IMSS with each other [2,3]. Davis and colleagues identified only one molecule (alcohol dehydrogenase 3, [GenBank:XM_650038]), where the expression profile was comparable on protein and RNA levels. This discrepancy between regulation at the transcript- and proteome level appears therefore to be a general characteristic of *Entamoeba*, indicating that this primordial eukaryote has a more complex way of expression regulation.

The strategy of identifying pathogenicity genes in *E. histolytica *by comparing pathogenic and non-pathogenic strains has already been used applied by other groups. They compared the pathogenic isolate HM-1:IMSS and the non-pathogenic isolate Rahman using microarray techniques [3-5]. A direct comparison of our results with all three microarray studies exhibits only poor overlaps. Of the 152 transcripts that were found in higher levels in the pathogenic isolate HM-1:IMSS in comparison to Rahman in microarray study performed by Davis and colleagues, only five are expressed in higher levels in the pathogenic cell line B (EhCP-A4 [GenBank:XM_651510], AIG family proteins [GenBank:XM_648115, XM_643009, homolog to HSP70 [GenBank:XM_648787], hypothetical protein [GenBank:XM_648447]). We identified only one gene expressed at higher rates in the non-pathogenic cell line A (hypothetical protein [GenBank:XM_644469]) among the 201 genes that are expressed in higher levels in Rahman compared to HM-1:IMSS [3].

Only 22 of the genes found to be differentially expressed between cell line A and cell line B were also found to be regulated in the study of Ehrenkaufer and colleagues and only eleven of them are regulated in both studies in the same direction [5]. These molecules include one of the two identified C2 domain proteins [GenBank:XM_650951], cell surface gp63 [GenBank:XM_647540], AIG family protein [GenBank:XM_648725] and eight hypothetical proteins [GenBank:XM_645291, XM_649962, XM_644469, XM_646066, XM_647129, XM_651187, XM_645139, XM_649961].

The low level of consensus between our study comparing two cell lines with the same genetic background and the remaining studies comparing two isolates with a different genetic backgrounds, leads to the assumption that the mechanism that determine the loss of virulence in Rahman differs from that observed for HM-1:IMSS cell line A.

Interestingly, only 19 of the investigated genes (0.3% of the predicted transcriptome) show a differential expression higher than five-fold between non-pathogenic cell line A and pathogenic cell line B. It can be assumed that genes where the level of respective transcripts differs to such an extent are involved in virulence. One of these molecules is a cell surface protease gp63 (EhMP8-2), which is transcribed at a more than 20-fold higher level in cell line A than in cell line B. In contrast to *ehmp8-2*, *ehmp8-1 *transcripts are found in similar abundance in both cell lines. In *E. histolytica*, neither of the gp63 proteases (EhMP8-1 and EhMP8-2) have been characterized. In Leishmania, the homolog, named leishmanolysin, occurs predominantly as a heavily-glycosylated protein that is attached to the outer membrane of Leishmaniapromastigates by a glycosylphosphatidylinositol anchor. It has been demonstrated that leishmanolysin plays a role in resistance of promastigotes to complement-mediated lysis and in receptor-mediated uptake of the parasite by phagocytic host cells [10]. It appears that most eukaryotes have homologs of this protein [11,12].

Transcripts of three genes coding for members of the Rab7 GTPase family (EhRab7D, 7E, 7G) were detected at much higher levels in the non-pathogenic cell line A. Rab proteins are essential for the regulation of vesicular trafficking in the endocytic and exocytic/secretory pathways of eukaryotic cells [13]. *E. histolytica *possesses more than 90 *rab *genes and therefore seems to be an organism with extremely diverse and complex Rab functions [14]. Rab7 in particular, has been described as one of the most important molecules involved in lysosomal biogenesis [15]. In different organisms it plays divergent roles in several distinct steps of endosomal or lysosomal trafficking [16]. *E. histolytica *encodes nine EhRab7 isotypes (EhRab7A-I), which show 40-65% identity to each other. It was shown that EhRab7A is associated with the post-Golgi compartment and is involved in the fusion of late endosomes. EhRab7B is localized to late endosomes/lysosomes and associated with the formation of lysosomes or the fusion to lysosomes. There is further evidence that all the EhRab7 isotypes are sequentially and coordinately involved in phagosome biogenesis [17]. In addition, we found that Vps35, is also transcribed at higher levels in cell line A (2.6-fold). Together with Vps26 and Vps29, it forms the amoebic retromer-like complex and functions as a EhRab7A-binding protein. This retromer-like complex is linked to the retrograde transport of putative hydrolase receptors from preparatory vacuoles and phagosomes to the Golgi apparatus. Nakada-Tsukui and colleagues showed that overexpression of EhRab7A caused enlargement of lysosomes and a decrease in cellular cysteine peptidase activity. The reduced cysteine peptidase activity was restored by co-expression of EhVps26. Thus, the EhRab7A-mediated transport of cysteine peptidases to phagosomes seems to be regulated by the retromer-like complex [18]. As mentioned above, phenotypic characterization of cell line A and cell line B showed a reduced cysteine peptidase activity in cell line A due to a reduced amount of mature EhCP-A1 and -A2 [8]. Although, it has been described that the interaction of EhRab7A with the retromer-like complex is specific as no association was observed with other isotypes such as EhRab7B or EhRab7D, an altered expression of molecules involved in vesicular trafficking may be responsible for the observed differences in cysteine peptidase activity [18].

We found two different transcripts of C2 domain-containing proteins in higher levels in the non-pathogenic cell line A. The C2 domain is a Ca^2+^-dependent membrane-targeting module found in proteins involved in membrane trafficking. Both molecules contain one N-terminal C2 domain and the C-terminal domains show high similarity to the P30 adhesin protein of *Mycoplasma pneumoniae *and to a hypothetical protein of *Paramecium tetraurelia *[GenBank:XP_001426443], respectively. P30 is a membrane-bound protein that is oriented with its N-terminus in the cytoplasm and its C-terminus on the cell surface, and is required for cytoadherence. The protein has three types of proline-containing repeats at its carboxy end [19-21]. Similar repeats were also found in the C2 domain-containing protein of *E. histolytica *[GenBank:XP_655299], giving rise to an overall 56% sequence identity. Nevertheless, the amoebic molecule seems not to be membrane-anchored, since it does not contain a signal sequence or a transmembrane domain. Additionally, the C-terminal region of the second C2-domain containing protein [GenBank:XP_656043] has a repetitive structure consisting of two main stretches.

In contrast to the transcripts described above, most members of the so far uncharacterized family of putative small GTPases, the AIG1 proteins, were found in higher levels in the pathogenic cell line B. At least 47 genes coding for AIG1 molecules are present in the *E. histolytica *genome. Quantitative real-time PCR analysis indicates that 18 of the 34 investigated *aig1 *genes are expressed at higher levels in cell line B. So far, the physiological relevance of these molecules is completely unknown in *E. histolytica*. They share partial homology to the GIMAP/IAN family molecules of vertebrates. Additionally, they have relatives in higher plants but not in most other well studied organisms, including bacteria, nematodes, and the amoeba *Dictyostelium discoideum *[9]. Nevertheless, *aig1 *genes are also present in the genome of *E. invadens*, *E. dispar *and *E. moshkovskii*, indicating that this family is conserved within the genus *Entamoeba *(data not shown). Structurally, all members of the family contain a GTP-binding cassette and several coiled-coil motifs [9]. In the *E. histolytica *homologs, only a part of the GTP-binding cassette is conserved and coiled-coil motifs are only predicted for some family members. GIMAP proteins are thought to be regulators of cell death in lymphomyeloid cells. It is suggested that the plant homolog AIG1 is involved in cell death regulation following self-defence responses to bacterial infection. Therefore, GIMAP proteins might be involved in self-defence machineries common to vertebrates and plants [9]. There are two further studies that discuss a link between pathogenicity and expression of *aig1 *genes. Davis and colleagues found *aig1 *genes expressed at much higher levels in HM-1:IMSS than in Rahman [3]. Additionally, the expression of *aig1 *genes was highly regulated in HM-1:IMSS trophozoites, obtained from a murine model of amoebic colitis. Trophozoites isolated from the cecal lumen of mice early in infection (day 1) showed either an unregulated or an increased expression of *aig1 *genes. Late in the infection (day 29), a decrease in the expression was observed [22]. Although this body of evidence points to an important role of *aig1 *genes for amoebic pathogenicity, their physiological role has to be determined.

## Conclusions

The availability of two syngenic *E. histolytica *cell lines (A and B), which differ constantly in their virulence, provides a powerful tool to identify pathogenicity factors of the causative agent of human amoebiasis. In addition to the proteomes, which have previously been compared between the cell lines, the differences observed in the transcriptomes were analyzed in the present study using microarray technology and quantitative real-time PCR. We identified a set of genes differentially transcribed between the non-pathogenic cell line A and the pathogenic cell line B. Most notably, various members of a family of putative *aig1 GTPase *genes are transcribed at higher levels in the pathogenic cell line B, whereas some *rab GTPase genes *are found in higher abundance in the non-pathogenic cell line A. The identification of transcription profiles unique for amoebic cell lines with specific virulent phenotypes, may aid the understanding of the transcriptional framework of *E. histolytica *pathogenicity.

## Methods

### *E. histolytica *cell lines and cell culture

In this study we used two genetically related *E. histolytica *cell lines (cell line A and cell line B). Both cell lines are derived from the *E. histolytica *isolate HM-1:IMSS as both were originally obtained from the American Type Culture Collection (ATCC) under the catalogue number 30459. HM-1:IMSS was originally isolated from a colonic biopsy of rectal ulcer from an adult male patient with amebic dysentry in 1967, Mexico City, Mexico. The monoxenic cultured HM-1:IMSS isolate was passed from Margarita de la Torre to Louis S. Diamond who adapted it to axenic cultivation. Thereafter, this axenically cultivated HM-1:IMSS isolate was transferred to the ATCC library. Cell line A was sent to us in 2001 by Barbara Mann, Charlotteville, University of Virginia, as a batch of cells from the same culture that was used for DNA preparation to sequence the *E. histolytica *genome [23]. Cell line B was obtained directly from ATCC in 1991.

The identical genetic background of both cell lines was confirmed by genotyping using tRNA-linked short tandem repeats from different chromosomal loci (tRNA primers D-A5+D-A3, N-K5, N+K3, R-R5+R-R3, S^TGA^-D5+S^TGA^-D3 and S-Q5+S-Q3) as described by Ali and colleagues [1,24].

Trophozoites were cultured axenically in TYI-S-33 medium supplemented with 10% adult bovine serum (ABS) at 35°C [25].

### Amebic liver abscess formation in gerbils

An amount of 1 × 10^6 ^amoeba trophozoites of *E. histolytica *cell line A or cell line B, respectively, in a volume of 100 μL were injected into the left liver lobe of eight week old male gerbils (Charles River) as previously described [26]. After seven days, sizes and weights of liver abscesses were determined. The experiment was repeated in intervals of 5-6 month during the last three years. Each time at least six gerbils were infected with cell line A or cell line B trophozoites, respectively. The cell population of both *E. histolytica *cell lines used for RNA extraction was tested in parallel for liver abscess formation.

### Microarray analyses

For transcription comparison, we used the *E. histolytica *70-base oligonucleotide two-channel microarray to analyze 6242 unique genes as described by Davis and colleagues [4].

RNA was isolated from approximately 5 × 10^6 ^pre-stationary phase *E. histolytica *cell line A and cell line B trophozoites grown in 75 mL culture flasks using Trizol reagent (Invitrogen) following the manufacturer's protocol. The total RNA was purified using the Qiagen RNeasy kit (Valencia, California) under modified protocol conditions without β-mercaptoethanol, including DNase treatment (Qiagen). RNA quantity and quality were obtained from an absorbance ratio at 260 and 280 nm. RNA quality was confirmed for each sample using an Agilent 2100 Bioanalyzer (Palo Alto, California) according to the manufacturer's instructions.

Cy3 or Cy5 labeled cDNA from cell line A and cell line B was cohybridized on one microarray. Altogether, four RNA samples (two biological replicates of each cell line) were competitively hybridized on four individual microarrays. Both Cy3 and Cy5 labeled cDNAs were created from each RNA sample using the Genisphere 3DNA array350 kit (Hatfield, Pennsylvania). Each pair of biological replicates was hybridized to two chips in which the Cy fluorescent channel was alternated in order to reduce dye-specific effects (dye swap). The primary and secondary cDNA hybridizations employed the 3DNA Array 350 kit for labeling, as previously described [4]. Slides were scanned on a ScanArray Express HT scanner (Perkin-Elmer, Boston, MA) to detect Cy3 and Cy5 fluorescence. Log_2 _ratios of cell line B versus cell line A samples were calculated, local background subtracted, and Loess normalized. Data analysis was accomplished with SQL scripts to calculate average and standard deviation for each transcript. Transcripts considered significant showed a 2.0-fold or more increase or decrease from control (cell line A), expression above the 10^th ^percentile, and a normalized standard deviation ratio (standard deviation/average) <1 to eliminate overly varying probes between biological and technical (dye-swapped and spotted in triplicate spotted) replicates.

### Quantitative real-time PCR

1 × 10^6 ^*E. histolytica *trophozoites were cultivated in 75 mL culture flasks for 24 h. The cells were harvested via chilling on ice for 5 min and sedimented at 200 × *g *for 5 min at 4°C. The cells were washed twice with PBS. For isolation of total RNA trophozoites were treated with TRIZOL reagent (Invitrogen) following the manufacturer's instructions. Extracted RNA was purified using the RNeasy mini kit (Qiagen) without β-mercaptoethanol and DNA was digested with DNase (Qiagen). cDNA synthesis was accomplished with SuperScriptIII Reverse Transcriptase (Invitrogen). In a final volume of 20 μL, 1 μg of RNase-free and DNase-treated total RNA was mixed with 5 × First-Strand buffer, 500 μM dNTPs, 500 nM OdT-T71 (5'-GAG AGA GGA TCC AAG TAC TAA TAC GAC TCA CTA TAG GGA GAT_24_), 2 mM DTT, 40 U RNaseOut (Invitrogen) and SuperScriptIII (200 U/μL). Reaction was incubated for 1 h at 42°C. For quantitative real-time PCR experiments sense- and anti sense primer were designed by amplifying approximately 120 base pairs of the accordant gene sequences (additional file 2).

Quantitative amplification was performed in a Rotor-Gene (Rotor-Gene 3000, Corbett) using RealMasterMix SYBR Green kit (Eppendorf). 1 μL cDNA was mixed with 2.5 × RealMasterMix/20 × SYBR and 5 pmol/μL of appropriate sense- and anti sense primer to a final volume of 20 μL. Amplification conditions were as follows: 35 cycles at 95°C for 15 s, 58°C for 20 s (1°C touch down for the first six cycles), 68°C for 20 s and an adjacent melting step (67°C-95°C). Two biological replicates were analyzed in duplicates. Analyzing relative changes in gene expression between cell line A and cell line B the 2^-ΔΔCT ^method, provided by Rotor-Gene software was used [27]. Accordingly, cell line A was representing the calibrator cell line and *actin *was chosen as normalizer gene. Efficiency ≥ 0.95 was empirically approved for selected primer and cDNA. Threshold for differentially expressed genes was set on 2.5.

## Competing interests

The authors declare that they have no competing interests.

## Authors' contributions

IB and ET conceived the study. IB coordinated the study and performed the data analysis together with LB and PD. IB, LB and ET drafted the manuscript. LB, PD, ST, JM, HL, and MT, carried out the laboratory component. All authors read and approved the final manuscript.

## Supplementary Material

Additional file 1 **List of genes differentially transcribed in *E. histolytica *HM-1:IMSS cell lines A and B identified by microarray analyses**. Using an microarray and analyzing two biological replicates, 87 gene transcripts were detected that show a two-fold or greater difference in expression between cell line A and cell line B. Out of these, 47 genes were significantly upregulated in the non-pathogenic cell line A and 40 genes were transcribed at significantly higher levels in the pathogenic cell line B. Click here for file

Additional file 2 **List of oligonucleotides used for real-time PCR**. Quantitative real-time PCR was used to confirm the differential transcription of 27 selected genes that showed at least a three-fold higher level of transcription in one or other cell line. Click here for file
