# Supplementary material for: Differences in the transcriptome signatures of two genetically related Entamoeba histolytica cell lines derived from the same isolate with different pathogenic properties
Source: BMC Genomics. 2010 Jan 26;11:63. doi: 10.1186/1471-2164-11-63 (PMC2823695; doi:10.1186/1471-2164-11-63)
Supplement: Additional file 1 — List of genes differentially transcribed in E. histolytica HM-1:IMSS cell lines A and B identified by microarray analyses. Using an microarray and analyzing two biological replicates, 87 gene transcripts were detected that show a two-fold or greater difference in expression between cell line A and cell line B. Out of these, 47 genes were significantly upregulated in the non-pathogenic cell line A and 40 genes were transcribed at significantly higher levels in the pathogenic cell line B. [file 1471-2164-11-63-S1.DOC]

**Additional file 1**

**Table:** List of genes differentially transcribed in *E. histolytica* HM-1:IMSS cell lines A and B identified by microarray analyses

| **Gene** | **GenBank**  **Accession-No. Gene** | **GenBank**  **Accession-No. Protein** | **Length**  **aa** | **Microarry results**  **(≥ 2.00)** | | **RT-PCR results**  **(≥2.50)** | | **Details** |
| --- | --- | --- | --- | --- | --- | --- | --- | --- |
|  |  |  |  | Cell line A | Cell line B | Cell line A | Cell line B |  |
| **Stress response** |  |  |  |  |  |  |  |  |
| Iron-sulfur flavoprotein | XM_650038 | XP_655130 | 195 | 2.09 |  | - | - |  |
| Fe-hydrogenase 2 | XM_647747 | XP_652839 | 504 |  | 2.44 |  | 3.22 |  |
| Methionine gamma-lyase | XM_647004 | XP_652096 | 389 |  | 2.38 | - | - |  |
| Hypothetical protein | XM_648787 | XP_653879 | 694 |  | 2.27 |  | 2.78 | 40% identity to heat shock protein 70 of *E. histolytica* (XP_648629) |
| **Trafficking/Targeting** |  |  |  |  |  |  |  |  |
| Vacuolar protein sorting 35 | XM_646067 | XP_651159 | 706 | 2.56 |  | - | - |  |
| Rab family GTPase | XM_646110 | XP_651202 | 207 | 62.00 |  | 743.45 |  | EhRab7E protein |
| Rab family GTPase | XM_651385 | XP_656477 | 190 | 14.50 |  | 50.00 |  | EhRab7G protein |
| Rab family GTPase | XM_650116 | XP_655208 | 264 | 2.23 |  | - | - | small GTPase EhRabD2 |
| Rab family GTPase | XM_646823 | XP_651915 | 204 | 25.37 |  | 125.00 |  | EhRab7D protein |
| C2 domain containing protein | XM_650207 | XP_655299 | 188 | 16.65 |  | 1000.00 |  |  |
| C2 domain containing protein | XM_650951 | XP_656043 | 389 | 6.62 |  | 33.35 |  |  |
| ENTH domain protein | XM_001913748  XM_645367* | XP_001913783  XP_650459* | 507 |  | 2.12 | - | - |  |
| **Transporter** |  |  |  |  |  |  |  |  |
| Major facilitator superfamily protein | XM_651348 | XP_656440 | 393 |  | 2.17 | - | - | SA**, 10 TMs*** |
| Sucrose transporter | XM_647746 | XP_652838 | 491 |  | 2.22 | - | - | SA, 11 TMs |
| **Signalling** |  |  |  |  |  |  |  |  |
| Ras family GTPase | XM_644490 | XP_649582 | 210 | 3.12 |  | - | - | Ras-related protein 3 |
| Ras guanine nucleotide exchange factor | XM_646718 | XP_651810 | 1214 | 2.00 |  | - | - |  |
| **Kinases** |  |  |  |  |  |  |  |  |
| Casein kinase II regulatory subunit family protein | XM_643682 | XP_648774 | 216 | 3.09 |  | - | - |  |
| **RNA/DNA metabolism** |  |  |  |  |  |  |  |  |
| 3' Exoribonuclease family protein | XM_646062 | XP_651154 | 223 | 3.14 |  | 5.34 |  |  |
| Activator 1 40 kDa subunit | XM_646064 | XP_651156 | 315 | 3.02 |  | 3.15 |  | DNA replication factor C |
| Hypothetical protein | XM_645298 | XP_650390 | 263 |  | 2.08 | - | - | RNA polymerase II transcription factor B subunit |
| **Cell cycle** |  |  |  |  |  |  |  |  |
| Rodhanase-like domain containing protein | XM_644512 | XP_649604 | 208 | 2.87 |  |  |  | M-phase inducer tyrosine phosphatase |
| Structural maintenance of chromosomes protein | XM_645549 | XP_650641 | 1023 |  | 2.38 | - | - |  |
| **Cell metabolism** |  |  |  |  |  |  |  |  |
| Alkyl sulfatase | XM_646063 | XP_651155 | 628 | 2.80 |  | - | - |  |
| Ornithine cyclodeaminase | XM_646653 | XP_651745 | 272 |  | 2.56 | - | - | L-proline biosynthesis |
| Carbonic anhydrase | XM_651594 | XP_656686 | 188 |  | 2.00 | - | - | Conversion of carbon dioxide to bicarbonate and protons |
| Alpha/beta fold family domain containing protein | XM_644468 | XP_649560 | 277 | 3.47 |  | 4.41 |  | Homology to alpha/beta fold family proteins and lysophospholipases  TM: 104-123, 143-165 aa |
| Metal dependent hydrolase | XM_646973 | XP_652065 | 218 | 2.14 |  | - | - | Metallo-beta-lactamase superfamily |
| Hydrolase yafV | XM_644478 | XP_649570 | 206 |  | 2.22 | - | - | Carbon-nitrogen family |
| Nucleoside diphosphate kinase | XM_649912 | XP_655004 | 211 |  | 2.08 | - | - | Exchange of phosphate groups between different nucleoside diphosphates |
| **Peptidases** |  |  |  |  |  |  |  |  |
| Cell surface protease gp63 | XM_647540 | XP_652632 | 662 | 21.44 |  | 70.58 |  | Peptidase M8 superfamily, EhMP8-2, SP**: 16 aa, TM: 598-620 |
| CAAX prenyl protease | XM_643678 | XP_648770 | 416 | 2.36 |  | 4.00 |  | Peptidase M48 superfamily  SA, 6 TMs |
| Cysteine proteinase | XM_651510 | XP_656602 | 311 |  | 2.56 | - | - | EhCP-A4, SP 20 aa |
| Cysteine proteinase | XM_652272 | XP_657364 |  | 2.67 |  |  |  | EhCP-A6, SP 15 aa |
| **Lectins** |  |  |  |  |  |  |  |  |
| Galactose-inhibitable lectin small subunit | XM_649244* | XP_654336* | 294 | 2.09 |  | - | - | SP: 17 aa, TM: 228-247, 254-276  Removed from NCBI |
| Gal/GalNAc lectin heavy subunit | XM_651089 | XP_656181 | 1286 | 2.05 |  | - | - | SP 15 aa, TM: 1226-1248 |
| **AIG family proteins** |  |  |  |  |  |  |  |  |
| AIG1 family protein | XM_648725 | XP_653817 | 335 |  | 14.29 |  | 100.00 | TM: 305 to 324 aa |
| Hypothetical protein | XM_648115* | XP_653207* | 80 |  | 12.50 |  | 100.00 | Removed from NCBI |
| AIG1 family protein | XM_645223* | XP_650315* | 407 |  | 12.50 |  | 4.76 | TM: 324 to 345 aa, 351 to 369 aa, Removed from NCBI |
| AIG1 family protein | XM_643009* | XP_648101* | 364 |  | 4.34 |  | 100.00 |  |
| **Hypothetical protein** |  |  |  |  |  |  |  |  |
| Hypothetical protein | XM_648456 | XP_653548 | 105 | 52.11 |  | 866.52 |  | 60% identity over 70 aa to annexin |
| Hypothetical protein | XM_645291 | XP_650383 | 863 |  | 14.28 |  | 50.00 |  |
| Hypothetical protein | XM_649962 | XP_655054 | 906 |  | 5.26 |  | 980.00 |  |
| Hypothetical protein | XM_646695 | XP_651787 | 2378 |  | 5.3 |  | 4.5 | Homology to Beige/BEACH domain containing protein |
| Hypothetical protein | XM_648447 | XP_653539 | 452 |  | 2.12 | - | - |  |
| Hypothetical protein | XM_651246 | XP_656338 | 2020 |  | 3.45 |  | 3.03 |  |
|  |  |  |  |  |  |  |  |  |
| Hypothetical protein | XM_644469 | XP_649561 | 438 | 3.52 |  | 5.71 |  | NHL repeat-containing protein |
| Hypothetical protein | XM_643681 | XP_648773 | 218 | 3.46 |  | 7.53 |  | Tetraspanin-like, (adhesion, motility, proliferation)  SA, 3 TMs |
| START domain containing protein | XM_649195 | XP_654287 | 219 | 2.03 |  | - | - |  |
| Hypothetical protein | XM_645260 | XP_650352 | 176 | 3.68 |  | 3.74 |  |  |
| Hypothetical protein | XM_644044 | XP_649136 | 297 | 2.03 |  | - | - |  |
| Hypothetical protein | XM_646065 | XP_651157 | 456 | 3.23 |  | - | - |  |
| Hypothetical protein | XM_642874 | XP_647966 | 233 | 3.06 |  | - | - |  |
| Hypothetical protein | XM_650614* | XP_655706* | 71 | 2.57 |  | - | - |  |
| Hypothetical protein | XM_647743 | XP_652835 | 135 | 2.54 |  | - | - |  |
| Hypothetical protein | XM_646719 | XP_651811 | 419 | 2.47 |  | - | - |  |
| Hypothetical protein | XM_643680 | XP_648772 | 158 | 2.36 |  | - | - |  |
| Hypothetical protein | XM_649953 | XP_655045 | 313 | 2.30 |  | - | - | Myristolation site predicted |
| Hypothetical protein | XM_647486 | XP_652578 | 224 | 2.28 |  | - | - |  |
| Hypothetical protein | XM_648775 | XP_653867 | 150 | 2.27 |  | - | - |  |
| Hypothetical protein | XM_646066 | XP_651158 | 589 | 2.23 |  | - | - |  |
| Hypothetical protein | XM_646972 | XP_652064 | 244 | 2.22 |  | - | - |  |
| Hypothetical protein | XM_643672* | XP_648764* | 359 | 2.13 |  | - | - |  |
| Hypothetical protein | XM_651312* | XP_656404* | 89 | 2.05 |  | - | - |  |
| Hypothetical protein | XM_647129 | XP_652221 | 222 | 2.00 |  | - | - |  |
| Hypothetical protein | XM_651187 | XP_656279 | 683 | 2.43 |  | - | - | TM: 307-329 aa  Homology to glycosyltransferases |
| Hypothetical protein | XM_649398* | XP_654490* | 148 | 2.36 |  | - | - | Homology to reverse transcriptases |
| Hypothetical protein | XM_646587 | XP_651679 | 660 | 5.52 |  | - | - |  |
| Hypothetical protein | XM_646717 | XP_651809 | 205 | 2.64 |  | - | - |  |
| Hypothetical protein | XM_651117 | XP_656209 | 378 | 2.64 |  | - | - |  |
| Hypothetical protein | XM_644818 | XP_649910 | 289 |  | 2.04 | - | - | TM: 256 to 278 aa |
| Hypothetical protein | XM_646245 | XP_651337 | 190 |  | 2.50 | - | - |  |
| Hypothetical protein | XM_651365 | XP_656457 | 1077 |  | 2.22 | - | - |  |
| Hypothetical protein | XM_651628 | XP_656720 | 410 |  | 2.00 | - | - |  |
| Hypothetical protein | XM_642828* | XP_647920* | 188 |  | 2.22 | - | - | Homology to HD hydrolase |
| Hypothetical protein T24C12.3 | XM_644703 | XP_649795 | 691 |  | 2.08 | - | - | SA |
| Hypothetical protein | XM_645139 | XP_650231 | 206 |  | 9.09 |  | 5.00 | SP: 20 aa, TM: 183-205 aa |
| Hypothetical protein | XM_646072 | XP_651164 | 195 |  | 2.22 | - | - |  |
| Hypothetical protein | XM_645300 | XP_650392 | 584 |  | 2.13 | - | - |  |
| Hypothetical protein | XM_648447 | XP_653539 | 452 |  | 2.13 | - | - |  |
| Hypothetical protein | XM_647137 | XP_652229 | 184 |  | 8.33 |  | 7.14 |  |
| Serine-threonine-isoleucine rich protein | XM_648869 | XP_653961 | 5069 |  | 6.25 |  | 3.85 | SP: 17 aa, TM: 4988-5010 |
| Hypothetical protein | XM_649025* | XP_654117* | 87 |  | 3.13 | - | - |  |
| Hypothetical protein | XM_643923 | XP_649015 | 889 |  | 2.33 | - | - | SP: 14 aa |
| Hypothetical protein | XM_649961 | XP_655053 | 161 |  | 2.27 | - | - | SA, TM: 854-876 aa |
| Hypothetical protein | XM_645369 | XP_650461 | 656 |  | 2.04 | - | - |  |
| Hypothetical protein | XM_647745* | XP_652837* | 51 |  | 2.00 | - | - | SP: 20 aa, TM: 28-50 aa Homology to stress response RCI peptide |
| Hypothetical protein | XM_647807 | XP_652899 | 904 |  | 2.00 | - | - | SP: 13 aa, TM: 871-893 aa |

*Removed from NCBI

**Prediction of signal anchor (SA) or signal peptide sequences using SignalP (http://www.cbs.dtu.dk/services/SignalP/)[ Bendtsen JD, Nielsen H, von Heijne G, Brunak S**: Improved prediction of signal peptides: SignalP 3.**0*. J Mol Bio*l 2004**, 34**0:783-795.]

*** Prediction of transmembrane domains (TM) using TMHMM Server (http://www.cbs.dtu.dk/services/TMHMM/)
