# Supplementary material for: Differences in the transcriptome signatures of two genetically related Entamoeba histolytica cell lines derived from the same isolate with different pathogenic properties
Source: BMC Genomics. 2010 Jan 26;11:63. doi: 10.1186/1471-2164-11-63 (PMC2823695; doi:10.1186/1471-2164-11-63)
Supplement: Additional file 2 — List of oligonucleotides used for real-time PCR. Quantitative real-time PCR was used to confirm the differential transcription of 27 selected genes that showed at least a three-fold higher level of transcription in one or other cell line. [file 1471-2164-11-63-S2.DOC]

**Additional file 2**

**Table.** List of oligonucleotides used for real-time PCR

| **Gene** | **GenBank**  **Accession-No.** | **5`Primer sequence** | **3`Primer sequence** |
| --- | --- | --- | --- |
| Fe-hydrogenase 2 | XM_647747 | GTGCTTATGGACCAGGGTGT | CAACCCATCTTCCTTCTGGA |
| Hypothetical protein | XM_648787 | TGTTATTCCTGTTGGTGGA | AGTGCTGCTCCTCTGGCTAC |
| Rab family GTPase | XM_646110 | CCGTGGTAGTGAATTGTTGTC | AACATTAGCACCATTGAGGAA |
| Rab family GTPase | XM_651385 | CTCACGAGCACTCAATGGAA | TTCCTTCACACCATTGACGA |
| Rab family GTPase | XM_646823 | GCAGGAAGACCAGCATTATT | GGTTATTGACGTATCGGTTG |
| C2 domain containing protein | XM_650207 | GGACGTTTTTCCAGGTGAAG | GACCTGGGAAACCATTCAAA |
| C2 domain containing protein | XM_650951 | CCAGGTTATCCTCAACAACC | GTTGAGCTGGTACTCCTGGT |
| 3' Exoribonuclease family protein | XM_646062 | CCAGACCGAGCAATTGTAA | AGGACAGTCCATTCAACACC |
| Activator 1 40 kDa subunit | XM_646064 | GGTCAACCAGGAATTGGTAA | CAGAAGCATTCAGTTCCAGA |
| Alpha/beta fold family domain containing protein | XM_644468 | ATAACCTTCACGTGTGCCTA | TGAATCGCAAGATGATTGAA |
| Cell surface protease gp63 | XM_647540 | ATTGCTGTCATTCCTGTGTG | TCACGAACTTCTTCTGCTTG |
| CAAX prenyl protease | XM_643678 | GGAATGCATTCTTTTCAGGA | TGTTACAATGTCCAACTTCATGT |
| Hypothetical protein | XM_648456 | GGTTATCCACCACAACCAAT | CCATAGGACCTGACATTCCTC |
| Hypothetical protein | XM_645291 | CATTCAACCAGCAATTGAAGT | TGATGATTCAAGTTATCCGACT |
| Hypothetical protein | XM_649962 | GCAGATCTTAATTGGTGCTTCAA | CTACCACCAGCCTCACCAAG |
| Hypothetical protein | XM_646695 | ATTGAATTGTGCTGCTCGTC | TCACCAATTCCAATAGTTCCTT |
| Hypothetical protein | XM_651246 | CATGTTCTGGAGACCCAACA | CAAATTGATCAACTGGCATATC |
| Hypothetical protein | XM_644469 | AATGAACCAACGAGTATTGA | TTCATATCTTCCTTGAAGGTCA |
| Hypothetical protein | XM_643681 | TGCTAGATCAGCTGAAGGAA | AATTGCACCAGCAACAATAA |
| Hypothetical protein | XM_645260 | CTACGAGAGTTCGCGTTAATA | GAAGATGAGTACAGACATGGTGA |
| Hypothetical protein | XM_645139 | AGATGGTGATGCTGTTGGAT | CCTGACTGTAACCATATCTCCA |
| Hypothetical protein | XM_647137 | CGTGATTATCAAGTGTGCGTTC | GCTTCAACATCAATCCAGTCTT |
| Serine-threonine-isoleucine rich protein | XM_648869 | TTGTTGTTGCAGCTCAAGATTT | TGGGGAAGACCAAACATCAC |
| AIG1 family protein | XM_643035 | ATTGGGTTTTACGGAGAAGGT | TAGTCAACCATTTGGTTCGTG |
| AIG1 family protein | XM_648725 | CCTCAAGCTGTGTTAGAAGAATC | TGACCTAGAGTTATCTTGTCCTG |
| AIG1 family protein | XM_643009 | TTGTGGACCAAGACCAACTAA | CCATGGTCCTTCTGATGATG |
| AIG1 family protein | XM_648115 | AAGAAAAAGCATCAGTCATA | GTTTTTCATTGCTCATAACT |
| AIG1 family protein | XM_643380 | GGGAATTGTCATAACATTGG | TTAACTCCAACAGCAACCTT |
| AIG1 family protein | XM_643637 | GGGTTTTTGGAAAACGGTAGT | CCTTACCAGCACCAAGAAGAG |
| AIG1 family protein | XM_643379 | TGGTGATTGGGAAATAGTCAA | CGCTCCTATTGCTCCTATTGT |
| AIG1 family protein | XM_644114 | AATTGAATGGGGAAGAGGAGT | TTCCCATTGATTCTGTTTCTTC |
| AIG1 family protein | XM_642923 | GAATGGGGAAGAGGAATAAC | TTTCCCATTGATTCTGTTTC |
| AIG1 family protein | XM_645021 | CCTTGGAACTACAGGTGATGG | TCCATATGAGCCAAGTGTTTG |
| AIG1 family protein | XM_643798 | AATGCTTTTAACATGGGCAAG | CGAGTTTCGTTTTTCTCTTCAA |
| AIG1 family protein | XM_649824 | TCTTTGGACCAAGAGGAAAAA | TGGTCCTTCTGATATGTTCCA |
| AIG1 family protein | XM_648140 | GGGTTTTACGGAGAAGGTGAT | TAGTCAACCATTTGGTTCGTG |
| AIG1 family protein | XM_645223 | TGGTCTTGCTAGTGGTGCTA | CAGGTGCTGTGATAAGTGGA |
| AIG1 family protein | XM_643164 | AATCCAGATGAAGGATGTGA | GGACTTTTTCATTTCCCTTT |
| AIG1 family protein | XM_643099 | ATTGGAAATACAGGTGATGG | TCCACTTGTTTCTTGTGTCA |
| AIG1 family protein | XM_643163 | ATTGGAAATACAGGTGATGG | TCCACTTGTTTCTTGTGTCA |
| AIG1 family protein | XM_643194 | GGAAATGGTAAAAGTTCACTTGG | TTCCATTTTCTCCAACAACATC |
| AIG1 family protein | XM_643099 | ATTGGAAATACAGGTGATGG | TCCACTTGTTTCTTGTGTCA |
| AIG1 family protein | XM_643163 | GCCGAGTAGAAGCTGATGAAA | CTCCATCACTTCCCATTCACT |
| AIG1 family protein | XM_643194 | GGAAATGGTAAAAGTTCACTTGG | TTCCATTTTCTCCAACAACATC |
| AIG1 family protein | XM_643240 | TGAAGGGTTACAAGGGATTA | TCTCACATTCAGTCCAAACA |
| AIG1 family protein | XM_643464 | TAATGGTGGTGCACTTTCTCA | CAAACATGCTTCCAAAAGTCA |
| AIG1 family protein | XM_643462 | GGAGGAATAGGAGATGGGAAA | TCGTCTCTTGTGTTTTTGGTG |
| AIG1 family protein | XM_642922 | TTGGTACTATTGCTGGTGCTG | TCATCATCGACTGCATCAACT |
| AIG1 family protein | XM_643102 | TTGCGTTAGAGCTGAAGGATT | CAAACGTGTTTCCAAATGTCTT |
| AIG1 family protein | XM_642959 | TGCTAAATGTTTTGGAGAAGGA | TTCCTTGTAATCCCTCAGCTC |
| AIG1 family protein | XM_643063 | AGGAAAAAGTATGGGGTGTTG | AACAGCACCACCAATGACAG |
| AIG1 family protein | XM_643097 | GCTAAAGAAACAACAGGAGATGAA | TACATGCCCATGTTAAAAGCA |
| AIG1 family protein | XM_644113 | AATTGGAGAAACAGGTGTTGG | TTCCAAAATAGCCAGCAACAT |
| AIG1 family protein | XM_644115 | TGGAAAAAGTATGGGGTGTTG | AACAGCACCACCAATGACAG |
| AIG1 family protein | XM_643463 | CGAAGAAACAACTGGAGATT | GTTCGTACCCAACTCAACAT |
| AIG1 family protein | XM_643100 | CTGGAAAAGAAGGTGTTGCTT | ACTACGAGCCCATGCTAAAAA |
| AIG1 family protein | XM_643195 | CCTTGGTGTTGGTGTTGG | AACAGCAGCAGCATTACC |
| AIG1 family protein | XM_643795 | CTTTCCCCATGTTCTTTGTTG | AAACTACGAGCCCATGCTAAA |
| AIG1 family protein | XM_643721 | TGGAGAAACAGGTACTGGTAAAA | TCACTTCTTTCTCCTTCTCCAA |
| AIG1 family protein | XM_648158 | AGCAAAGACCAACGAAATTA | CTGAATCCGGACTACCACTA |
